# Update on Treatment Options for Advanced Bile Duct Tumours: Radioembolisation for Advanced Cholangiocarcinoma

**DOI:** 10.1007/s11912-017-0603-8

**Published:** 2017-06-28

**Authors:** Pavan Najran, Angela Lamarca, Damian Mullan, Mairéad G. McNamara, Thomas Westwood, Richard A. Hubner, Jeremy Lawrence, Prakash Manoharan, Jon Bell, Juan W. Valle

**Affiliations:** 1 Department of Radiology, The Christie NHS Foundation Trust, Manchester, UK; 2 Department of Medical Oncology, The Christie NHS Foundation Trust, Wilmslow Road, Manchester, M20 4BX UK; 3 Division of Molecular and Clinical Cancer Sciences; Institute of Cancer Sciences, Faculty of Biology, Medicine and Health, University of Manchester, Manchester Academic Health Science Centre (MAHSC), Manchester, M13 9PL UK

**Keywords:** Intrahepatic, Cholangiocarcinoma, Liver radioembolisation (RE), Advanced, Treatment, Chemotherapy

## Abstract

Cholangiocarcinoma is a rare form of gastrointestinal cancer with a poor prognosis. Patients often present with biliary obstruction or non-specific abdominal pain, and a high proportion of patients have advanced disease at initial diagnosis. The goal of this review is to discuss treatment options for patients with advanced bile duct tumours focusing on radioembolisation (RE) and its impact on overall survival. RE provides a therapeutic option for patients with unresectable cholangiocarcinoma. However, although systemic chemotherapy has demonstrated a survival benefit in randomised controlled trials, there is limited supporting evidence for the use of RE in this setting. Studies are mostly limited to single-centre, small cohorts with variable outcome measures. Additionally, patients included in these studies received a variety of previous therapies including chemotherapy, surgery or alternative intra-arterial therapy; therefore, a true assessment of overall survival benefit is difficult.

## Introduction

Biliary tract cancers (BTCs), including cholangiocarcinoma, ampulla of Vater and gallbladder cancer, are rare cancers with a poor prognosis [1•]. The incidence is increasing, mainly due to a rise in diagnosis of intrahepatic cholangiocarcinoma [2–4]. BTCs account for just 3% of all gastrointestinal malignant tumours in adults [5]. Cholangiocarcinomas arise from epithelial cells of bile ducts and are subdivided according to the location of the primary tumour into intrahepatic, hilar and distal cholangiocarcinoma [1•].

The peak incidence of BTC is between the ages of 50 and 70 years; cholangiocarcinoma is more frequent in males, while females have higher incidence of gallbladder cancer [5]. Medical conditions associated with chronic gallbladder or biliary tract inflammation (e.g. gallstones, gallbladder/biliary duct polyps, primary sclerosing cholangitis, chronic biliary/gallbladder infections, congenital anatomical abnormalities of the biliary tract) are known risk factors for developing BTCs [6–8].

The prognosis for patients with BTC is poor: the 5-year survival (5-YS) is 5–15% when considering all patients [9, 10]. The stage at presentation directly impacts on survival; the 5-YS is 50% for stage I, 30% for stage II, 10% for stage III and 0% for stage IV [11, 12]. Patients diagnosed with BTCs may present with biliary obstruction, due to local infiltration and occlusion of the biliary tract, mainly in patients with distal or hilar cholangiocarcinoma. In contrast, patients diagnosed with intrahepatic cholangiocarcinoma are more likely to present with non-specific right upper quadrant pain, which may delay diagnosis. Therefore, the majority of patients (60–70%), and particularly those patients diagnosed with intrahepatic cholangiocarcinoma, will be diagnosed at an advanced stage of disease, when curative approaches are not available [13].

In addition, even in patients treated with curative resection for localised stages, the relapse rate is high [11, 14, 15]. Uncontrolled studies have explored adjuvant strategies in BTC patients following potentially curative surgery [16–18]; however, the results of prospective randomised studies are awaited (ACTICCA-1 [clinicaltrials.gov registration number NCT02170090], BILCAP [NCT00363584] and a study using gemcitabine/oxaliplatin in the adjuvant setting [NCT01313377] [19]).

## Treatment Options for Advanced Disease

The most frequently used treatment modality is chemotherapy, particularly in the presence of systemic (usually extrahepatic) disease. The current reference regimen is cisplatin and gemcitabine based on the pivotal phase III study showing an advantage in overall survival (OS) from this combination compared to gemcitabine alone (11.7 vs. 8.1 months, respectively; *p* < 0.001) [20•]. These findings were confirmed in a Japanese randomised phase II study (BT22 study) [21] (see Table 1). Other regimens using fluoropyrimidines (such as 5-fluorouracil), gemcitabine and other platinum agents, either in combination or as monotherapy [22–24], have been reported. However, their efficacy has not been confirmed in randomised phase III studies.

**Table 1:** Summary of some clinical trials exploring the role of cisplatin/gemcitabine systemic chemotherapy in BTCs

Trial	Author, reference	Type of trial	Type of chemotherapy	Number of patients	Patients with ICC	Line of treatment	Median OS (months)	Median PFS (months)
ABC-02	Valle et al. [20•]	Randomised phase III	Cisplatin + gemcitabine vs. gemcitabine	410 (204/2016)	80	First-line	11.7 (95%CI 9.5–14.3) vs. 8.1 (95%CI 7.1–8.7)	8.0 (95%CI 6.6–8.6) vs. 5.0 (95%CI 4.0–5.9)
BT22	Okusaka et al. [21]	Randomised phase II	Cisplatin + gemcitabine vs. Gemcitabine	83 (41/42)	29 (14/14)	First-line	11.2 (95%CI 9.1, 12.5) vs. 7.7 (95%CI 6.1–11.0)	5.8 (95%CI 4.1–8.2) vs. 3.7 (95%CI 2.1–5.3)
GERCOR	André et al. [22]	Phase II^a^	Cisplatin + gemcitabine	56 (33/23)	29 (16/13)	First-line and second-line^a^	15.4/7.6	5.7/3.9

*CI* confidence interval, *ICC* intrahepatic cholangiocarcinoma, *OS* overall survival, *PFS* progression-free survival

^a^The GERCOR clinical trial divided patients into two prognostic groups: the first one (group A) was good prognosis patients (*n* = 33) with Eastern Cooperative Oncology Group performance status 0–2, less than 2.5 times upper limit of normal (ULN) total bilirubin and without previous chemotherapy treatment. The second group (group B) patients were poor prognosis: greater than performance status 2, bilirubin above 2.5 times ULN or progressive disease to a previous chemotherapy schedule

Following progression on first-line chemotherapy, approximately 15% of patients are suitable for further chemotherapy, mainly due to rapidly progressive disease and worsening performance status [25]. In addition, the magnitude of benefit, if any, from second-line chemotherapy is unknown [26•]. Active symptom control (e.g. by biliary stenting and antibiotics, as appropriate) is considered the standard of care in some countries as the benefit suggested by small prospective and retrospective studies [25, 27–29] has not been confirmed in prospective studies. The ABC-06 study (NCT01926236; [19]) is a randomised phase III trial which compares the combination of oxaliplatin and fluorouracil (FOLFOX) against active symptom control alone in patients with advanced BTC following progression on first-line cisplatin and gemcitabine; recruitment is ongoing.

Unlike patients with gallbladder cancer or distal cholangiocarcinoma, patients with intrahepatic cholangiocarcinoma may have liver-only (or liver-predominant) disease; in such cases, a liver-directed approach (e.g. RE) may be considered following systemic therapy [30•, 31].

### Intrahepatic Cholangiocarcinoma: Morphology

Cholangiocarcinoma presents with a variety of different morphological features secondary to variable cellular components, such as fibrous stroma, contributing to variable imaging appearances [32]. Intrahepatic cholangiocarcinoma is subclassified into three main types according to its features: mass forming, infiltrative and intraductal [33]. Identifying key imaging features of these subtypes aids in interpretation of imaging, prognostic factors and avenues for surgical and non-surgical management [34].

Mass-forming cholangiocarcinoma is characterised as a homogenous well-demarcated lesion with an irregular margin. The lesions commonly possess a capsular rim due to compression of the adjacent parenchyma which also results in biliary ductal dilatation and may precipitate patient presentation due to jaundice [35]. Due to the large fibrotic component of this subtype, capsular retraction and late centripetal enhancement are also common features [36]. Appearances on magnetic resonance imaging (MRI) are similar to those of computerised tomography (CT) with delayed contrast enhancement and obliteration of the portal vein without visible tumour thrombus [37]. Uncommon features of this subtype include hypervascular enhancement suggestive of a well-differentiated tumour [38]. Additionally, there are several mimics for mass-forming lesions including hepatocellular carcinoma (HCC) with cirrhotic stroma, sclerosing HCC and combined HCC and cholangiocarcinoma; therefore, these have to be considered particularly on a background of cirrhotic liver disease [39].

The periductal infiltrative subtype is uncommon; key features include a branchlike thickening of the intrahepatic ducts with or without ductal obliteration [40]. Imaging using CT and MRI shows periductal enhancement with ductal dilatation if found peripherally. However, this subtype is seen more commonly in the hilar region [41]. Differentials for the periductal subtype include lymphangitic metastasis and peribiliary cysts. Distinguishing features for cholangiocarcinoma include ductal dilatation and localised lobar disease [42].

The intraductal subtype has a variety of features such as ductal ectasia. The most common CT and MR imaging features are of diffuse ductal dilation with either a polypoid or plaque-like mass with post-contrast enhancement [43]. This form of cholangiocarcinoma is characterised by slow growth and a more favourable prognosis than its counterparts [44].

### Hepatic IAT

The use of locoregional therapies in patients with intrahepatic cholangiocarcinoma is becoming more established [45]. Different methods of intra-arterial therapy include bland embolisation, trans-arterial chemoembolisation (TACE), the use of drug-eluting beads and radioembolisation.

Liver intra-arterial therapy is a well-established locoregional therapy used in the treatment of patients with localised HCC, and its use in randomised trials has demonstrated a survival benefit [46]. The use of intra-arterial therapy (IAT) in cholangiocarcinoma is not as well documented; although studies have suggested a survival benefit, this evidence is limited to single-centre or retrospective studies [47] (see Table 2 for a summary of the most relevant studies). Additionally, the published studies use variable outcome measures to assess treatment benefit. Therefore, a true comparison of IAT with alternative treatments is limited.

**Table 2:** Summary of the most relevant studies examining the use of RE therapy and its impact on overall survival

Study design	Year	Reference	Type of intra-arterial therapy	Patients (*n*)	Median overall survival
Retrospective multicentre review	2013	Hyder et al. [46]	TACE vs. DEB vs. TAE vs. RE	198	TACE 13.4 months vs. DEB 10.5 months vs. TAE 14.3 months vs. RE 11.3 months
Systemic review	2014	Al-Adra et al. [30•]	RE	298	15.5 months
Prospective study	2009	Saxena et al. [48]	RE	25	9.3 months
Prospective single centre	2012	Hoffmann et al. [49]	RE	33	22 months
Prospective single centre	2013	Rafi et al. [50]	RE	19	11.5 months
Prospective single centre	2014	Mouli et al. [51]	RE	46	Solitary lesion 14.6 months vs. multifocal lesions 5.7 months

*TACE* trans-arterial chemoembolisation, *DEB* drug-eluting beads, *TAE* bland embolisation, *RE* radioembolisation

HCC derives its blood supply from the hepatic arterial vessels, rather than the portal supply, providing an intrinsic advantage to the use of IAT [52]. In contrast, cholangiocarcinoma is not as hypervascular, and therefore, direct IAT may not provide as much benefit [53] and so is a potential limitation to its use in this setting.

Intra-arterial therapy is delivered directly into the hepatic arterial branches, and RE can be performed in a single step or as a two-stage procedure. The initial stage is to perform catheter angiography, establishing the arterial anatomy and identifying any aberrant vessels supplying the liver, as well as the gastric and gastro-duodenal arteries. Traditionally, the right gastric and gastro-duodenal arteries are occluded and a test dose of technetium (^99m^Tc)-microaggregated albumin (MAA) is delivered at the treatment point. Occlusion of the local right gastric and gastroduodenal arteries (GDA) is performed to prevent reflux of particles into these vessels and subsequent radiation enteritis. A ^99m^Tc MAA single-photon emission computed tomography (SPECT) CT is then performed to assess for extra-hepatic uptake and to calculate the lung shunt. Utilising the angiogram and the MAA SPECT CT data, a dose calculation can then be performed for the yttrium-90 (^90^Y) microsphere treatment.

After the catheter angiography and MAA SPECT CT step, the procedure involves delivering the treatment particles (either in two separate doses into the right and left hepatic arteries depending on the distribution of disease or less commonly as a single dose to the entire liver when injected via the hepatic artery proper). Delivery of the particles in two separate doses, or in some cases three, allows more accurate dose calculation compared to one large-dose delivery.

The choice of particles for yttrium delivery is between two established glass and resin particles. Glass particles (TheraSpheres, MDS Nordion, Toronto, Ont, Canada) individually contain greater radioactivity; therefore, there is a reduced number of particles delivered per treatment compared to resin particles (SIR-Spheres; Sirtex Medical, Sidney, NSW, Australia), which individually contain a lower dose and so a larger number are delivered per treatment [54]. A summary of the particle characteristics is provided in Table 3. Although a study has suggested a minimal survival advantage with the use of glass particles over resin [56], such evidence is limited with other publications reporting no difference [57].

**Table 3:** Summary of the characteristics of the two particles used in RE therapy

Parameter	Resin	Glass
Trade name	SIR-Spheres	TheraSpheres
Diameter	22 ± 10 μm	32 ± 10 μm
Specific gravity	1.6 g/dL	3.6 g/dL
Activity per particle	50 Bq	2500 Bq
Average number of microspheres per administered activity	40–80 million	1.2–8 million
Material	Resin with bound yttrium	Glass with yttrium in matrix

Source: [55]

### Considerations When Developing a Treatment Algorithm for the Treatment of Patients with Intrahepatic Cholangiocarcinoma

Surgical resection, where possible, is the cornerstone of therapy; however, in patients presenting with advanced disease, this is not appropriate [58]. Systemic chemotherapy has an established, albeit modest, survival benefit [20•]. However, there is limited evidence supporting the use of RE in this patient subgroup. According to the National Comprehensive Cancer Network (NCCN) guidelines, chemotherapy is a recommended treatment option for patients with unresectable intrahepatic cholangiocarcinoma; there is a reference to the use of locoregional therapy but no specific reference to the use of RE (https://www.nccn.org/professionals/physician_gls/pdf/hepatobiliary.pdf). Additionally, comparative studies between systemic chemotherapy and RE are limited to single-centre, non-randomised studies or in the retrospective setting [30•, 46, 59]. The use of RE in other hepatic malignancies such as colorectal liver metastases has been evaluated with a phase III clinical trial, randomising patients to first-line chemotherapy alone vs. chemotherapy and RE [60]. The results showed no improvement in overall PFS at any site; however, there was a significant delay in progression within the liver [60]. A systematic review by Al-Adra et al. reported a mean OS in patients with unresectable cholangiocarcinoma treated with RE therapy of 15.5 months [30•]. The reported survival is favourable in comparison to the previously reported median OS of 11.7 months in patients with advanced BTC treated with cisplatin/gemcitabine chemotherapy [20•]; therefore, within the limits of the evidence available, RE may be a potential treatment option.

A limitation to a number of the studies comparing various treatments includes the selection of a heavily pre-treated population; therefore, the true benefit of the therapy in question cannot be accurately tested [59]. Also, various methods have been used to calculate treatment response or to predict outcomes with a number of studies assessing treatment response using either Response Evaluation Criteria in Solid Tumours (RECIST) or modified RECIST (mRECIST) [61]. Camacho et al. concluded that the use of mRECIST criteria accurately predicted OS whereas RECIST did not in patients with advanced BTC [62].

Pre-treatment prior to RE treatment (i.e. with systemic chemotherapy) may mask the true benefit of RE. A study performed by Haug et al. reported prolonged survival in patients treated with RE who were chemotherapy-naïve. This was also reported in a study performed by Ibrahim et al. [59]; however, these conclusions must be interpreted with caution as the studies include small patient cohorts and are therefore prone to selection bias.

The volume of disease and liver function are key factors to consider when assessing patients for suitability for RE. Radioembolisation delivers a focal radiation dose to the liver parenchyma; therefore, those patients with large volume disease or underlying liver dysfunction will be at risk of liver failure, called radioembolisation-induced liver disease (REILD).

A consistent factor in predicted treatment outcome with RE therapy is patient performance status with a number of studies consistently showing that patients with better performance status have a greater OS post-RE treatment. A study performed by Hoffmann et al. reported an OS benefit in patients with an Eastern Cooperative Oncology Group (ECOG) performance status of 0 versus 2 (29.4 months vs. 5.1 months) [49].

When considering treatment options, tumour volume, liver function and performance status are important factors to consider in addition to previous treatment received. A multidisciplinary (MDT) approach will allow assessment of the essential factors to predict survival benefit and assess appropriate treatment.

## Discussion and Conclusions

Radioembolisation is a novel modality of therapy for patients with metastatic liver disease and unresectable primary liver cancer. It is licensed in the UK for the treatment of patients with metastatic colorectal cancer without significant extrahepatic disease and in patients with cholangiocarcinoma where chemotherapy has failed (http://sirt.org.uk/sirt-uk.php).

The OS benefit appears to be favourable when compared to the reported literature on the use of non-IAT. The experience of its use at The Christie, within the limitations of the cohort size, supports a favourable OS advantage when compared to published survival figures for patients treated with alternative therapy acknowledging the retrospective setting.

Alternative intra-arterial therapy has been used in the treatment of patients with advanced BTC, including TACE and bland embolisation; however, there are no directly comparable randomised studies, and it is, therefore, difficult to compare survival statistics between these different locoregional treatment options. Additionally, patients treated with RE commonly have had previous treatment, which makes it difficult to assess the true benefit of RE alone.

Although there are no randomised trials, the published data is promising. The reported OS for patients diagnosed with unresectable cholangiocarcinoma treated with cisplatin/gemcitabine combination is 11.7 months [20•]. An important factor reiterated in a number of studies is the impact of patient performance status on OS, with improved OS for patients with better performance status [45]. This is therefore important when considering a patient for RE and should be an essential element to any treatment algorithm. The underlying disease will impact on patient survival. Patients with primary cholangiocarcinoma who are suitable for radioembolisation will have liver-only disease and therefore will be stage I to III. However, those patients with metastatic colorectal cancer would be, by definition stage IV, and are therefore more likely to have a poorer outcome. Due to an element of stage shift, patients will have variable survival depending on the underlying disease.

The variability in the patient cohort size, patient performance status, disease burden and previous treatment may contribute to discrepancies in OS figures reported in a number of studies. Although results are promising, larger, multicentre randomised trials comparing RE with alternative IATs and systemic chemotherapy are required to demonstrate whether RE provides a true benefit.

A study investigating the use of the cisplatin/gemcitabine combination +/− RE as first-line treatment for patients with unresectable intrahepatic cholangiocarcinoma (SIRCCA) is currently listed on clinical trials.gov (NCT02807181) but is not yet recruiting [19]. An additional listed study recruiting in Hong Kong aims to study the benefits of sequential administration of RE followed by standard chemotherapy for the treatment of inoperable intrahepatic cholangiocarcinoma (NCT02167711) [19]. The benefit of RE versus TACE for the treatment of patients with cholangiocarcinoma (NCT01798147) is also being evaluated. These studies should provide more guidance on the use and sequencing of this therapeutic modality in the treatment of patients with advanced BTC.
